# Students’ perceptions of schools’ influence on the leadership self-efficacy of adolescent girls: religious and secular post-primary schools in Israel

**DOI:** 10.3389/fpsyg.2025.1488270

**Published:** 2025-03-13

**Authors:** Shenhav Perets, Nitza Davidovitch, Eyal Lewin

**Affiliations:** ^1^Department of Education, Ariel University, Ariel, Israel; ^2^Department of Middle East Studies and Political Science, Ariel University, Ariel, Israel

**Keywords:** leadership development, teenage girls, post-primary school, self-efficacy, case study

## Abstract

The aim of this study is to evaluate the perceived effect post-primary school has on female teenagers’ leadership self-efficacy according to their own perceptions. The study employs social cognitive theory and focuses on the teenagers’ personal social experiences and perceptions regarding the way leadership is taught in their schools. The research is based on 26 in-depth interviews among teenage girls attending middle and high schools in the public education system in Israel. The teenage girls attend secular state education schools and religious all-girl state education schools (Ulpana). The findings indicate four major ways in which self-efficacy can be developed. The religious all-girl schools seem to promote leadership self-efficacy more effectively than secular mixed-gender schools, primarily by mastering leadership experiences, social modeling, and social persuasion of leadership ability.

## 1 Introduction

People who hold leadership positions are expected to facilitate the development of a direction for an action or behavior, coordinate with others in support of this direction, and engage with and motivate others to accomplish this direction (Day and Dragoni, 2015). In a challenging, uncertain, and changing world that requires embodying several leadership roles in life, adolescence seems to be the period in which teenagers must be engaged in leadership-developing experiences as part of a process of building their character and preparing them for leadership positions as adults (Larson et al., 2019). Their identity, especially their leader identity, forms at this age (Hoyt and Kennedy, 2008). Although any teenager can become a leader, given the right educational conditions, experiences, and awareness of his or her ability to be one (Van Linden and Fertman, 1998), there is a significant difference between boys and girls in this context. Girls often have low self-esteem compared to boys in secondary school, and the gap between them expands in high school (Eccles et al., 1993). Adolescent girls also seem not to take risks, avoid competition, not always challenge traditional gender stereotypes, be concerned with body image, and worry more than boys their age (Harter, 1999). Shapiro et al. (2015) found that socialized gender roles are already evident by middle school and impact adolescent girls**’** career aspirations. As far as leadership is concerned, studies point out that girls are more exposed than boys to socialization processes that make them understand they are less compatible for leadership roles, they get fewer messages from their environment that they have leadership abilities, and they are less likely to be viewed by others as leaders (Archard, 2012a). The work of Susan Murphy, Chair in Leadership at the University of Edinburgh, and her colleagues are notable in this regard (see, e.g., Murphy and Johnson, 2011; Murphy and Reichard, 2011; Hoyt and Murphy, 2016; Darling et al., 2006; Ensher and Murphy, 1997; Johnson et al., 2008). Therefore, examining adolescent girls**’** educational conditions as a foundation for their empowerment and encouraging them to become leading women has become an important subject in youth leadership studies over the last thirty years.

While leadership development can take place throughout an individual**’**s lifespan, schools may need to implement early interventions to address the leadership gap between genders. This gap arises from two interconnected factors: gender socialization and gender bias (Fulton et al., 2019).

Gender socialization constitutes a developmental process through which individuals acquire and internalize gender roles, which are shaped by culturally defined expectations for gender-specific behaviors (Fulton, 2017). These expectations are frequently grounded in stereotypes—broadly held assumptions regarding the attitudes, characteristics, and behavioral patterns attributed to males and females. The process of gender socialization commences early in life and can significantly influence a girl**’**s self-efficacy, perceptions of leadership, political ambitions, overall confidence, and aspirations regarding career paths and skill development (Haber-Curran and Sulpizio, 2017). Furthermore, girls encounter a second challenge stemming from a bias in leadership perceptions that tends to favor boys; such biases can be perpetuated by both genders and, at times, by parents (Weissbourd et al., 2015). This prejudice against female leadership may be elucidated by competitive dynamics among girls, a deficiency in confidence and self-esteem (which they may project onto their peers), or prevailing stereotypes that characterize girls as excessively emotional or expressive (Weissbourd et al., 2015).

This finding is not unexpected, as the characteristics typically associated with traditional leadership are more congruent with societal perceptions of male attributes than with those of females. The processes of gender socialization and bias have tangible consequences. Gender expectations can restrict the choices available to girls and adolescents during middle and high school, thereby potentially influencing their higher education, career trajectories, and earning capacities (Bian et al., 2017). In numerous cultures, females are stereotypically linked with communal traits, such as nurturing and expressiveness, while males are associated with agentic traits, including competitiveness and rationality. Given these stereotypes, it is unsurprising that women are more likely to pursue professions that emphasize communal qualities (e.g., homemaker, nurse), whereas men are drawn to careers that prioritize agentic traits (e.g., physician, manager). Consequently, professions typically associated with women tend to offer lower salaries and diminished prestige.

Considering these biases, it is crucial not only for girls to be given access to leadership development opportunities and diverse career options but also that deliberate measures are implemented to establish programs specifically designed to dismantle these barriers. In this context, it is posited that universal leadership programming, which does not account for gender-specific challenges, may be inadequate in addressing the unique obstacles encountered by girls and adolescents. Consequently, scholars in this field assert that targeted leadership programs for girls can enhance their self-confidence, expand their career possibilities, and diminish the influence of gendered messages (Fulton et al., 2019; Shapiro et al., 2015).

Gender plays a significant role in the experiences provided by leadership development. Boys and girls experience different challenges, developmental processes, and identity formation processes that influence their self-confidence and self-efficacy with regard to leadership abilities (Archard, 2013). Women face a number of challenges and barriers in the process of assimilating the identity of a leader and translating a belief in being able to lead into a motivation to lead (Bandura, 1997). On the other hand, the effects of leadership self-efficacy on motivation to lead seem to be stronger for males (Hoyland et al., 2021). Since men’s and women’s behavior is affected not only by psychological factors but also by socio-structural factors, we should try to understand it and the mechanisms that produce a certain behavior by using an integral approach (Bandura, 1999). Therefore, when exploring the process of leadership development among teenage girls, we must be aware of the uniqueness of their emotional and behavioral state.

## 2 Leadership and leader development

As explained by Day (2000), the distinction between developing leaders and developing leadership is an important one. Leader development focuses on developing individual leaders and typically emphasizes individual-based knowledge, skills, and abilities associated with formal leadership roles. Leadership development, on the other hand, is a more complex process than one concerned solely with individual leader development. It concentrates on a process of development that inherently involves multiple individuals (Day et al., 2014). Teams and organizations can enhance their capacity to engage in leadership due to leadership development processes. “Shared leadership could be considered as one form of collective leadership capacity as it involves greater numbers of team members engaged in leadership than a solo leader” (Day et al., 2021, p. 3). Therefore, in accordance with the given definitions, this study deals mainly with collective group leadership and not with individual leader development.

### 2.1 Leadership development at school

School is the first formal organization that most people experience, and as they grow up, children gradually understand the importance of this organization in their lives (Montgomery and Kehoe, 2016). School might also be the earliest social establishment that influences our organizational behavior (Karagianni and Montgomery, 2018), and it is a place where children form their sense of intellectual efficacy, enabling them to participate in large-scale activities in society (Bandura, 1997). The essence of leadership is taught, among other things, by assigning students to key leading roles at school. The experience of fulfilling roles has been shown to help youth build strategic thinking and responsibilities (Larson and Hansen, 2005). Research has also indicated how trusting program leaders enhanced the youth’s active engagement in developmental processes (Griffith and Larson, 2016; Griffith et al., 2018; Larson et al., 2019). By presenting role models such as teachers, administrators, visitors, and outstanding students, schools enhance students’ motivation to participate in demanding roles, an important factor in their development (Sperandio, 2010).

During the last few years, programs that intend to turn young people into future active citizens have become central to nation-building plans of governments all over the world (Khan, 2022). Studies have shown that such school leadership programs have a positive effect on adolescents’ self-esteem, confidence, self-efficacy, constructive social interaction, and awareness of their ability to lead (Karagianni and Montgomery, 2018; Chan, 2003; Wong et al., 2012). School has an essential role in character-building and creating a solid foundation for youth leadership (Wallin, 2003; See and Arthur, 2011; Agboola and Tsai, 2012; Diggs and Akos, 2016; Zurqoni et al., 2018) and significantly influences young women’s beliefs and understanding of leadership while providing opportunities to develop and practice leadership (Sperandio, 2000; McNae, 2011). Therefore, research focusing on girls’ leadership development at school is highly important. While being the main arena for obtaining knowledge and technical and social skills, schools also have the ability to enhance girls’ resilience (Mampane, 2014) and motivate young women, given the right programming and environment (Wong et al., 2012; Mann et al., 2015; Eva et al., 2021).

Although the literature on developing teenage girls’ leadership and how they perceive their leadership abilities has grown in the last twenty years, not many studies focus on girls’ personal leadership perception in a school context. Examining the effectiveness of formal lessons, non-formal programs, and interventions schools carry out to develop leadership skills and traits can determine crucial factors in self-efficacy, as can exploring aspects of school that are less noticeable or aimed directly at enhancing leadership, such as the school’s atmosphere, members of staff as role models, actual leadership experiences, and peer influences (Bussey and Bandura, 1999; Eva et al., 2021; Tusianah et al., 2021). Such factors are investigated in this study.

Furthermore, not enough research has been conducted on the leadership development of Israeli teenage girls as a specific field of interest, especially in the public school context. Female youth leadership ventures in Israel have been investigated elsewhere (Sheffer, 2023) but not in relation to specific leadership programs. There are also various research works in Hebrew about gender equality and its implementation in Israeli high schools (Gilad et al., 2010; Herzog and Valden, 2011; Gur Ziv, 2013; Avgar, 2017; Pedagogical Administration, 2020). However, not many explore girls’ leadership self-efficacy in general, still less through the specific theoretical framework of social cognitive theory.

The purpose of this research is to examine teenage girls’ perceptions of the effect that post-primary school has on their leadership self-efficacy while analyzing their leadership experiences during their studies. It focuses on self-efficacy as a key element in the leadership development process and compares two groups of students’ perceptions of their school’s influence on this issue: adolescent girls who study in the secular public education system and adolescent girls who study in the religious public all-girls schools called Ulpana. Focusing on Israel, this research examines the extent to which, according to the students’ accounts, post-primary schools develop the perception and abilities of leadership among female teenagers. The comparison is made in the context of the secular and religious educational sub-systems.

### 2.2 Leadership, self-efficacy, and the social cognitive theory

Involving teenage girls in school programs, lessons, and activities concerning leadership and leadership development is insufficient to explain their leadership potential to them or to allow them to become aware of it. Their self-perception as young women who are able to lead is assembled through a process of character building. One of the crucial stages in this process is improving their perceived leadership self-efficacy. “Unless people believe that they can produce desired effects by their actions, they have little incentive to act or to persevere in the face of difficulties” (Bandura, 1999, p. 28). Having a goal, committing to its achievement, and believing the desired outcome can be completed is a major cognitive mechanism that enhances motivation.

A study conducted by Tusianah et al. (2021), which analyzed 20 published papers concerning self-efficacy, indicated it could be increased by factors such as being treated fairly, taking part in activities and programs that are organized and designed according to participants’ characteristics, and having manageable levels of stress, a sense of anticipation, and good feelings in general. On the other hand, feeling discriminated against, having difficulties with overcoming problems, lacking support, and having burdensome conditions can damage self-efficacy. Wood and Bandura (1989) found people of high efficacy to be more resourceful and adaptable, which makes them more likely to manage their environment more effectively and productively.

Leader self-efficacy is defined as follows: “A leader’s judgments of their capabilities to organize and execute courses of action required to attain designated types of leadership outcomes” (Rehm and Selznick, 2019, p. 53). In this study, though, “leadership self-efficacy” will serve as a more accurate term since it examines teenage girls still in their leadership development process. Leadership self-efficacy is associated with the level of confidence in the knowledge, skills, and abilities a person has in leading others (Hannah et al., 2008). Since the end results people prepare for depend largely on their belief in how well they can perform in given situations (Bussey and Bandura, 1999), people will likely perform as leaders for as long as they believe they can actually do it. According to Day et al. (2008), leader self-efficacy, leader self-awareness, and leader identity are vital elements in the process of developing leadership. In other words, if a person views himself or herself as a leader and maintains a high level of efficacy to perform as a leader when needed (Van Knippenberg and Hogg, 2003), then he or she is more likely to demonstrate enhanced effectiveness as a leader in comparison to people with limited self-awareness, a weak leader identity, and lower levels of leadership self-efficacy (Hannah et al., 2008). This is why it is both relevant and important to focus on the development of leader self-assessment as a direct outcome in the leadership development process (Day and Dragoni, 2015).

In recent years, social cognitive theory (SCT) has become a common approach for analyzing leadership development (O’Connell, 2014; Yeow and Martin, 2013). SCT provides a theoretical framework for research on the development of adolescent girls as leaders through three key areas: the learning process in detail, drawing on the reciprocal nature of the adolescent girl and her environment; how adolescent girls learn through the modeling process; and how adolescent girls develop the self-efficacy to engage in leadership (Bandura, 1999; Bussey and Bandura, 1999). The SCT frame combines a new perspective for understanding adolescent girls’ leadership self-efficacy, interpreting the processes used in leadership development programs, and forming a link to leadership development literature on a much larger scale.

Eva et al. (2021), in their review of 108 articles from the last 20 years, emphasized the lack of a consistent theoretical framework in research into adolescent girls’ leadership development and recommended research to be rooted in SCT. The fragmentation of the literature across fields may be related to this contradictory theoretical discussion. Nevertheless, SCT-based research offers a theoretical framework that examines three aspects: the learning process adolescent girls experience through the ongoing interaction between identity, behavior, and their environment (Bandura, 1989), learning through the modeling process (Wood and Bandura, 1989), and developing the self-efficacy to engage in leadership (Bussey and Bandura, 1999).

This last element of SCT is the primary concern of this research, which focuses on four factors that affect leadership self-efficacy among girls (Bussey and Bandura, 1999):

1. Mastery Experiences: When a person achieves success through graded experience, he or she builds a growing sense of self-efficacy. When they fail, self-efficacy declines.
2. Social Modeling: Knowledge, skills, and abilities are not only acquired through direct experience; it is also important to consider the enormous effect that modeling has on self-efficacy. Modeling, in this sense, is a way of judging and observing other people’s actions and consequences to form values, styles of thinking, and behavior patterns.
3. Social Persuasion: A person is more likely to believe he or she is able to succeed when other people express their faith in their capabilities.
4. Physical and Emotional State: When stress and depression are reduced and people feel strong and emotionally and physically satisfied, their belief in their capabilities intensifies.

In general, our focus on Bandura’s work stems from his emphasis on the importance of observational learning, imitation, and modeling in behavior development. SCT integrates cognitive processes with social influences, highlighting how individuals learn from their surroundings and the vital role self-efficacy plays in influencing behavior. Bandura’s research on the interplay between behavior, environment, and personal factors underpins the principles of SCT (Devi et al., 2022; Kauffman and de la Fuente, 2023; De la Fuente et al., 2022).

### 2.3 Leadership education in Israel

This study seeks to understand whether post-primary school affects adolescent girls’ leadership self-efficacy in Israel (according to their own perceptions) and, if so, in what way. However, we should first consider that girls do not all attend the same educational system. There are two main forms of education for the Jewish population in Israel from preschool to high school, and the differences between them are quite significant.

The Jewish education system in Israel receives funding from the central government, supplemented by additional financial resources from local municipal authorities. This system comprises two principal sub-sectors: state secular and state religious education, each designed to serve families ideologically inclined to choose either secular or religious schooling for their children. The majority of Jewish pupils (73.2 percent) attend secular state education institutions, which include both boys and girls in the classrooms, while 26.8 percent attend religious state education institutions, which are divided into all-girls and all-boys schools.

In most subjects, the students in the two sub-sectors are exposed to similar learning content and curricula, except for areas where ideological considerations necessitate distinct approaches. Consequently, the curricula for subjects such as Hebrew, English, mathematics, science, geography, and physical education are largely comparable across both sectors. However, subjects that address ideologically sensitive content, such as literature, history, art, and particularly religious and heritage education, are specifically tailored to meet the unique requirements of each sub-sector.

The ethos and vision of the state secular education system in Israel emphasize the instillation of values, morals, and good citizenship, all of which are crucial to the development of a distinct Israeli culture. This perspective posits that the primary objective of secular schools is to foster a value system that encompasses aims, goals, cultural awareness, knowledge, skills, and emotional intelligence that contribute to the establishment of norms characteristic of a modern or post-modern society. Consequently, values education is regarded as a critical component of the educational process, focusing on the cultivation of the culture, values, beliefs, attitudes, and emotional well-being of students. In this context, the term ‘culture’ within the state secular school system effectively replaces the term ‘religious belief’ in state religious schools.

The philosophy of secular education in Israel is founded on three fundamental concepts that underpin the religious and heritage education provided in state secular schools: acquired truth, which is contingent upon the pursuit of knowledge; freedom and originality of thought; and modernity, alongside contemporary humanistic perspectives of the world.

Parents opt to enroll their children in this educational stream due to their secular perspectives and lack of religious commitment. They aim to provide their children with a comprehensive and balanced secular education that prioritizes academic achievement alongside principles of citizenship rooted in democracy, equality, social harmony, humanism, and universalism, all without any reference to religious observance or lifestyle. Students are encouraged to pursue their studies to achieve high levels of academic performance, enabling them to attain quality matriculation and graduation credentials. Consequently, religious and heritage education within this sector focuses on exploring Jewish history, culture, and identity while intentionally omitting discussions of the practical observance of religious tenets. Furthermore, the curriculum emphasizes values that promote social cohesion and encompasses the citizenship values of democracy, equality, social harmony, humanism, and universalism, which are central themes in religious and heritage education in this context.

The state religious education sector is distinguished by the intrinsic rationale underlying its existence. Its primary objective is to cultivate and deepen students’ religious beliefs, behaviors, and understanding of Jewish tradition while also fostering a sense of responsibility toward the community and the needs of others. The study of religion in state religious schools is additionally anticipated to engage with challenges that may arise when religious beliefs intersect with contemporary moral and ethical considerations. Moreover, religious and heritage education within this system is structured to reinforce Jewish religious beliefs, traditions, and moral values, thereby preparing students to embrace the normative expectations of society.

Parents who select the state religious education sector for their children do so primarily because they believe that the central focus of their children’s education lies in the religious emphasis integrated into all aspects of cognitive and affective development within this sector. In state religious and heritage education, there is a strong emphasis on faith and knowledge-based instruction, where religious and heritage education encompasses a variety of subjects taught from a deterministic religious perspective. In addition to the general subjects taught in state education, many hours in religious schools are devoted to studying the Bible, Toshba, the Talmud, religious laws, and traditional Israeli thought. In some general subjects, such as literature and history, they study a unique curriculum adapted to the values of Jewish religious education. Religious schools also have different requirements for compulsory subjects in order to obtain a matriculation certificate: increased study of the Bible and studying the Jewish Talmudic oral literature. This curriculum is designed to promote adherence to religious precepts and commandments while ensuring academic success in other core subjects required for matriculation examinations at the conclusion of students’ educational journeys (Katz, 2017, 2018, 2021).

The scholarly literature examining leadership training within religious contexts primarily focuses on the influence of religion itself and the impact of religious factors within the broader religious educational framework on the cultivation of leadership qualities among individuals (McMaster, 2013; Lumby and Ruairc, 2021). In these academic works, leadership development is analyzed predominantly from a religious standpoint (Spencer and Lucas, 2019). This focus persists even in comparative studies that investigate diverse settings, such as Catholic, Islamic, and Greek Orthodox institutions (Striepe et al., 2014). The significance of faith as a pivotal element in fostering leadership among youth is apparent even in studies that explore experiences within religious environments that are not strictly educational (Freeman, 2024). However, this study diverges from that trend.

This paper refrains from addressing theological issues or the influence of religious values on leadership training. Instead, it focuses on students’ leadership training experiences in two distinct yet comparable environments: a mixed-gender secular educational system and an all-girls religious institution.

Generally, students in Israel get knowledge of and practice in leadership through various channels at the same time. Some leadership programs specifically state that their purpose is to develop leadership, while others do so indirectly by emphasizing features such as responsibility, social involvement, project leadership, and personal empowerment. The Society and Youth Administration, a department of the Ministry of Education, supervises programs for young guides as early as the eighth grade for volunteer students who express an interest in organizing holiday camps for children and field trips all over the country. This department is also in charge of student councils in schools, in cities, and at a national level. Participating in the three-year “Personal development and social involvement” program, established in 2014, is required of every high-school student. Its purpose is to encourage the realization of teenagers’ potential to become independent, resilient, full of self-worth, and sensitive to other people’s needs.

In addition, the principals and staff of each and every school have the opportunity to combine several leadership programs in the curriculum, such as “young mediators” groups, “empowerment groups,” and initiative programs. Some invite lecturers and experts from external programs who specialize in leadership to talk to the students. Leadership is also taught formally within different lessons: history and civics lessons, for example, include discussions about historical or political figures through the ages. Some lessons in literature are dedicated to a character displaying a leadership personality. Bible lessons very often discuss great biblical leaders and the way they ruled and made crucial decisions. All classes in post-primary schools in Israel are obligated to have a lesson called “educational lessons” at least twice a week. These lessons are about values, life skills, communication, and getting to know one another informally. Some of these lessons are meant to encourage leadership potential among the students.

## 3 Research questions

In order to understand the processes of leadership development among teenage girls in Israel and the way in which schools contribute by enhancing leadership self-efficacy, this paper sets out to answer the following research questions:

*Q1*: In what way do post-primary schools enable teenage girls’ leadership experiences?

*Q2*: In what way do teenage girls perceive people in their school environment to be leadership mentors or role models?

*Q3*: Do teenage girls receive messages at school from meaningful figures regarding their ability to lead?

## 4 Methodology

Educational research should be obligated to reveal the apparent and concealed processes in educational organizations in order to explain the formation of student identity and expose practices defining positions of power to certain groups. This is essential because education includes a combination of values and beliefs and the social construction of both the individual and society (Shalsky and Arieli, 2016). Youth leadership is also shaped by the social context within which it functions; therefore, a quantitative approach in general, and in-depth interviews in particular, is the ideal method to explore social construction. In-depth interviews are a means for a descriptive study that aims to comprehend the views and perspectives of informants in specific inquired circumstances (Brinkman, 2018). It is especially important when female subjects are concerned. As Dorothy E. Smith (1992) explained, referring to “women’s standpoint,” women act, see, and speak based on their experience as women. “At the root of in-depth interviewing is an interest in understanding the lived experience of other people and the meaning they make of that experience” (Seidman, 2006, p. 9). Therefore, this research uses a qualitative method, focusing on the meanings girls give to their leadership experiences at school.

### 4.1 Research participants

In order to extract these meanings, in-depth data was gathered mainly by interviews conducted with 26 teenage girl students aged 13 to 18. Fourteen of them study at 11 different secular state education schools in Israel. These formed the first research group. The other 12, forming the second research group, study at six different religious all-girl state education schools called Ulpana.

Since all the participants shared a common general education experience, they were more likely to provide information that could help this research examine the relevant concepts. Although the students attended different middle and high schools in the public education system in Israel (either religious or secular), each one participated in one or more leadership programs in her school or at least joined lessons and lectures revolving around the subject. The sample groups share a number of characteristics, including their ages, the fact that they are female students enrolled in post-primary institutions under the Ministry of Education’s guidance with regard to the lessons and values instilled in them, and their similar experiences with regard to some of the leadership programs run by the Israeli educational system. The differences between secular and religious schools are related to the curriculum of religion and to the fact that the Ulpana is an all-girls school while state education is gender-mixed with no boarding school conditions. Noteworthy, while the majority of Ulpana schools operate as boarding institutions, a subset functions as partial-boarding schools. The participants in this study attended the latter type of institution, returning to their homes each day.

One ought to be aware of a certain difference between the two samples. The Ulpana is a boarding high school for girls who wish to combine religious studies with the regular curriculum; it is the equivalent of the Yeshiva high school constructed particularly for girls (Ben Shetreet and Woolf, 2019). Consequently, tuition fees are higher in the Ulpana, which might indicate an inherent difference between the two investigated populations. However, the specific institutes investigated in this paper are not necessarily prestigious ones, and the researchers therefore found it appropriate to make comparisons.

This study used a snowball sampling method, which is often used in qualitative research because it allows for a deeper understanding of complex, subjective experiences within a specific context. The people referred to by initial interviewees are often considered to be similarly knowledgeable or relevant to the study, which aligns with the goal of obtaining in-depth insights (Noy, 2008). The initial interviewees were sampled by meeting teenage girls and their parents in various cities in Israel on different occasions, hearing about individuals during preliminary interviews, or asking for interviewees on social media, such as WhatsApp. As the research progressed, no additional participants were interviewed when it was evident that no new information regarding this study’s purpose was being added; in other words, the criteria for sufficiency and saturation had been obtained (Seidman, 2006).

### 4.2 Data collection

The interviews were conducted over Zoom, an internet application that enables face-to-face interaction, and lasted approximately one hour. The interviewer who conducted all the interviews and coding process is the primary researcher in this study. She is an educator and researcher of education, gender, and society, specializing in youth leadership in formal and informal educational settings. Each interview was carried out while the interviewee was at home and at a time that was convenient for her so that she would be provided with a sense of security and be encouraged to speak openly and honestly (Dixon, 2015).

The subject of the study and its sociological and social implications were explained to the interviewees and their parents well in advance. Parents’ written consent was received prior to the interview, and it was clarified to both them and their daughters that participating in this study was optional and not mandatory. It was also emphasized that their personal details would remain confidential and their identity would be anonymized.

Since this study focuses on the interviewees’ words and sentences, it was important to let their speech be given center stage. Therefore, the interviews were guided by a sharing approach (Devault, 1990; Reinharz, 1992), and the information shared by the researcher was limited to general details so as not to impose her point of view on the interviewee (Dixon, 2015). Active listening was implemented as an element of the interview approach, facilitated by identifying the mechanisms in the comments and statements made by the interviewee in response to the interviewer’s questions. The fact that the interviewer was a woman enabled closeness and intimacy despite the generation gap between her and the interviewees (Edwards, 1993; Mullen and Tuten, 2004). Furthermore, genuine attempts were made in order to produce a non-hierarchical environment throughout the interview (Oakley, 1981) and establish trust (Dixon, 2015).

The core of the interview revolved around a series of questions written in advance about the way the interviewee describes herself in general, the role school plays in her life, her school’s references to leadership, what leadership means to her, and how she perceives her abilities, her school’s atmosphere, leading figures at school, and her social status at school. LSE components were an essential element of the issues posed, serving as the basic foundations upon which this study was formed. During the interviews, more layers of knowledge about these girls’ experiences were revealed, leading to many other questions for clarification.

### 4.3 Data analysis

This study’s data analysis method comprised three stages (Schmidt, 2004). First, categories were formed by marking similarities and differences between the interviews. Second, passages in the transcripts were related to these categories by a coding process, performed by the authors of this paper. It should be noted that multiple coding and cross-validation procedures were not employed in this study. The third stage involved gathering all passages concerning a specific theme in a table that divided secular and religious schools in a way that enabled the researcher to identify patterns and observe similarities and differences between the two groups.

During this long process, the classification and organization of the information revealed various themes regarding self-efficacy in relation to leadership development. In addition, the process identified mechanisms that shape the process of establishing a leadership personality, which is one of the most important aims of the study.

## 5 Findings

The interviews brought four notable ways in which self-efficacy can be developed to the fore, each highlighting the differences between the two groups investigated: mastery experiences of leadership both during and after school; social modeling transmitted by meaningful adults or peers; social persuasion of their ability to lead from key figures at school; and the teenage girl’s physical and emotional state.

### 5.1 Mastery experiences

The interviews revealed mastery experiences in both groups. Almost every girl stated that she actively acted or is currently acting as a leader. Nevertheless, the groups differ in the circumstances in which these experiences occur. 78.5% of the girls who study at secular schools reported taking leadership positions almost exclusively during formal school hours (leading projects and assignment groups) and as part of their school’s leadership programs (initiated in school or brought into school by a private association); only 14.2% reported taking leadership positions after school as well. On the other hand, 66.6% of Ulpana girls talked about having leadership experiences at school (holiday and ceremonial projects, for example), and 100% reported leadership experiences after school hours (guiding younger children at youth centers and organizations unrelated to their school).

#### 5.1.1 Girls attending secular schools

Public education in Israel sometimes promotes leadership development through outsourcing. Programs are carried out in school by non-profit organizations or private associations, and students are welcome to participate if they want to and if they feel they can dedicate themselves to the process. Student 2 talked about her experience in a project conducted in her school by Unistream, an organization that offers programs to schools all over Israel that enable the youth to acquire professional skills and entrepreneurial tools in order to motivate them to come up with new solutions and ideas and promote their future success:

*We had Unistream this year. I was elected CEO. I had to supervise my friends, making sure they were doing their job right. I had to submit our nomination to the committee. I wrote polls for our work and filled out forms. Yes, I think it went really well, and I found it really easy to lead the way. I shared my ideas and my decisions with the group. It went well in the social aspect. I think it really boosted my skills as a leader.*

As well as participating in external leadership programs, the interviewees expressed mastering leadership experiences on many occasions as part of their learning processes:

*I took command of my civics project this year. My group was a little tired. I tried to lift them up. So instead of dividing the work and telling them, “You do this, and you do that,” I tried making them work together and help each other while making sure no one was working harder than anyone else… It made me realize I’m capable of leadership on a small scale, and maybe, later on, I can try to do it on a bigger scale.* (Student 13)

Students 2 and 13, as well as the other girls studying in the non-religious schools, shared their experiences of mastering leadership at school and emphasized how much they liked it. They used phrases such as “taking command,” “boosted my skills,” “felt like I was in charge,” “helped my team reach their goal,” “taking responsibility,” and “it made me realize I can do it,” all of which indicate their awareness of their leadership potential based on experience. As far as self-efficacy is concerned, it seems that the positive perspective these girls have of their experiences as leaders contributes to their belief in themselves.

#### 5.1.2 Girls attending Ulpana

As mentioned earlier, Ulpana girls have more opportunities to play a leading role during their adolescent years due to their widescale participation in youth movements and comprehensive involvement in project management tasks at school. Student 24, who volunteered as a guide at a program for special needs children in a community center, explained how this position made her feel truly able to lead:

*I really felt like a leader and like I have the ability. I remember that at the first meeting, the manager of the community center told me, “I forgot to tell you, you will be speaking in front of the parents today too to explain everything about the program.” [I thought,] I haven’t even finished 11th grade, how am I going to do that? Eventually, the parents sat around me in a circle, and I did it. It felt really good. I felt like they were counting on me. I presented exactly what I wanted to happen during the program, and we even scheduled another meeting for the end of the year. At that point, I can honestly say I knew I could do it.*

In another part of the interview, she talked about mastering leadership in school programs:

*We usually team up for projects. When I feel I’m not so good at a subject, I let others lead. But when I love the subject and know I can do it, I say, “Girls, let’s go! Let’s work together now and not waste time.” I motivate them to act, especially if I like the project.*

Student 21 talked about being able to perform in leadership positions both inside and outside school:

*These things usually happen in the branch [of the youth movement] or at the Ulpana. At the branch, as a guide, I’m able to make the little kids do stuff, get them to listen to me. And also, at the Ulpana, two months ago, we were really late getting a school play together. I was on the master board. There are a lot of committees, but the master board kind of monitors everything. I felt I had the ability to take responsibility for things and make them happen. Make sure everything works.*

Mastery experiences for Ulpana girls occurred both inside and outside school. Every Ulpana girl who was interviewed (as well as all their friends, so they claimed) participated in a Zionist youth movement, either Bnei Akiva or Ariel, and therefore was experiencing and mastering leadership in this context as well. In addition, these girls gain leadership experience at school, but unlike the girls in the secular education system who are provided with diverse programs and projects, almost no external programs are held in the Ulpana. Leadership experiences in those institutions mainly fall within the scope of ceremonies and special occasions, initiated and promoted by the staff or the students themselves.

Self-efficacy, therefore, is intensified through mastery experiences in both groups. However, the fact that more Ulpana girls act as leaders more frequently than girls in the secular education system (since most of them are guides in youth organizations) suggests that they have greater self-efficacy in their ability to lead.

### 5.2 Social modeling

Besides mastering leadership experiences in and out of school, as opposed to mainly at school, Ulpana girls also seem to have the advantage of being motivated by more social models, that is, people in formal leadership positions. An analysis of the interviews of girls who study in the public education system reveals that they either receive negative messages from their environment with regard to female leadership or have no social modeling for leadership whatsoever.

#### 5.2.1 Girls attending secular schools

Listening to teenage girls studying in the public education system made it very clear that they have almost no social model who embodies knowledge, skills, and leadership strategies they can relate to in their immediate environment as far as school is concerned. Sometimes, observing their peers’ failure to lead instilled disbelief in their ability to do this kind of activity (Bussey and Bandura, 1999).

*I think that the ones who do the most behind the scenes are girls, but the boys are the ones who eventually get the credit. They get to be the so-called “leaders.” The mind behind every joint assignment is a girl’s, but the boys take all the credit. This is what always happens. Behind every male leader are a few women who did all the work.* (Student 1)

*Looking at the girls in my class, and there are many of us, none of them seems to me to be someone that will be able to manage in life. At least in the state they’re in right now. I won’t be able to manage as well because I don’t think we’re given any tools.* (Student 8)

The interviewees expressed disbelief in themselves—both as a group and as individuals—when they observed their peers. Being able to take credit for their actions and achievements and handle obstacles during adulthood is considered to be difficult. Therefore, these girls are likely to learn that they will later be confronted with the same difficulties and continue to fail to accomplish tasks. Their perception of a lack of substantial social models at school intensified when they were questioned about members of staff. In fact, only six (42.8%) pointed out a specific member of staff as a role model. When Student 9 was asked for an example of a teacher whom she perceives as a leader, a role model, or someone to look up to, she said:

*We are very close to our teachers. We sit together during recess, and we laugh during classes. Even if someone did something wrong, everything is forgiven, everything is solvable.*

Student 14, in response to the same question, said:

*My student counselor. She always makes sure that we’re okay. Sometimes she takes us out of class for personal conversations. I also like my English teacher. The way she teaches us.*

Students 4 and 14, like most of the interviewees in this research, perceive teachers as caregivers, similar to what they consider characteristic of a parental approach: being nurturing, giving, kind, and reassuring. Although the questions specifically stressed leadership as the key element they should identify when discussing their teachers, they did not mention any leadership skills, attitudes, or charisma. Some even expressed their utter disappointment with their teachers:

*Most teachers just go there to teach. To give homework. To test us. Some teachers go beyond that. They teach the subjects the same as they teach leadership. Our school’s motto is “Leadership and identity.” Something like that. So it promotes leadership. But it depends on the teacher. Some of the teachers just don’t care about it, and some do.* (Student 3)

*Not all teachers set a good example for the students, so it’s problematic. But it really depends on the teacher. One teacher can be very meaningful to you and teach you all sorts of values and things you’ll remember, and then another teacher just comes to do her job. It really depends on the person.* (Student 6)

Using phrases like “just go there to teach” and “just comes to do her job” when speaking about their female teachers demonstrates how the gender knowledge these girls absorb from their immediate environment is negative. Since girls pay more attention to modeling, in and out of school, and are more likely to transform the message they get from their environment into actual performance based on the gendered behaviors they observe, their self-efficacy regarding leadership will likely be negatively affected (Wood and Bandura, 1989).

#### 5.2.2 Girls attending Ulpana

Evaluating the Ulpana girls’ interviews reveals a fundamentally different approach regarding social modeling. Not only do they appear to be surrounded by many more social models that reinforce the connection between leadership and femininity, but they also seem strongly motivated by others to practice leadership themselves. For example, Student 15 talked about the influence her friend has on her:

*I have a very good friend who really deals with a lot of stuff and … she doesn’t see herself handling a lot of things. She sees herself as a regular person. I learn so much from her, like how to be more responsible, to take care of things, to help others. I don’t know if she’s aware of the fact that I learn from her, but I do, and I know there are girls who are learning from me, so…*

Interestingly, Ulpana girls talked a lot about the positive effect(s) of female figures surrounding them in school. Eleven (91.6%) expressed genuine admiration for their female teachers, student counselors, and female guides. Observing these significant educators, watching how they conduct themselves in school, and listening to what they say inside and outside the classroom caused the interviewees to be impressed by their schools’ members of staff.

*I really love my student counselor. Just recently, she celebrated 40 years of her career. She is a powerful, strong woman with a stirring personality who helps everyone. She has the character of a leader and does everything with a kind heart. If I take one thing from school with me, it’s her.* (Student 17)

*Our social coordinator at the Ulpana is a figure who has all the possible roles at school. She’s in charge of so many things! If she leaves, eight people are going to have to take her place… She is there all the time, and we consider her to be kind of a friend. She knows how to run things and makes sure everything she wants will happen. She’s very stubborn and builds good connections with people so they will help her when she needs their help. A real leader.* (Student 23)

Other Ulpana girls, referring to their teachers, used phrases like “they are always with us,” “I admire my teachers for working so hard and still being attentive,” “she had so much patience,” “she pushes me to learn,” “she really cares about how we feel,” “she’s my mentor,” and “amazing personality.” This indicates the meaning these teachers have in the interviewees’ world and how much they respect them and value their work.

### 5.3 Social persuasion

Social persuasion (others expressing their belief in one’s capabilities) is one of the most significant elements in building self-confidence and a strong sense of belief in oneself. This category seems to be a weak spot for girls in the public secular education system.

#### 5.3.1 Girls attending secular schools

Five out of 14 (35.7%) interviewees talked about experiences in which a teacher, most commonly a homeroom teacher, tried to convince them that they have what it takes to lead. As Student 13 explained:

*I feel that my homeroom teacher empowers me a lot. She has told me many times that in any field of knowledge, especially the social sphere, I can contribute to the school, take part in the decision-making process, and make a difference.*

Interestingly, Student 13 was the only one who stated that a teacher approached her specifically in order to motivate her to take a leadership position. The other four students indicated a more general approach:

*I don’t think teachers choose anyone in particular. They usually don’t say, “Take this group and lead them.” It happens during the group’s work on a project. … No one spoke to me about it, but I do hear our teacher talking to all of us all the time, saying how much he believes in us. He tells us we can do anything. He is very encouraging and gives us a lot of confidence.* (Student 11)

When the other nine girls answered questions regarding social persuasion concerning their ability to lead, they often made comments that varied from a definite “no” to “not recently” or “not from anyone at school.” Interviews with Ulpana girls, on the other hand, portrayed a different picture.

#### 5.3.2 Girls attending Ulpana

Ten out of 12 interviewees (83.3%) confirmed being persuaded about their ability to lead by significant figures in their school. Six of them talked about a teacher who is close to them, either their homeroom teacher or one who teaches a subject they are particularly interested in, and spends many hours with them during the day.

*My homeroom teacher has a lot of personal conversations with us. She even does it in her own free time. She always makes us feel very comfortable sharing. When I was having doubts about whether I should be in charge of the school production in the Purim festival, she told me, “You can do this! You’re gonna make it!”* (Student 23)

*My teacher told me I had leadership qualities several times in the context of my community service plans after I graduate, which is teaching the Torah and Judaism to non-religious children.* (Student 25)

The most surprising finding among this group appeared when the remaining four interviews were analyzed. These girls mentioned the support they were given either by peers and friends who study with them at school or by a member of staff who was not a teacher, principal, or student counselor but a female guide who is actually almost their own age. The guide’s role is a common feature of all the Ulpana schools in Israel. This young woman is aged between 18 and 20, is an Ulpana graduate herself, and works as a volunteer at school as part of her national service—an alternative voluntary national service for religious Jewish girls who choose not to serve as soldiers in the Israeli army. Apparently, these female guides have a substantial effect on the Ulpana girls:

*I think maybe my guides, because I had my best personal relationship with them. They knew how to point out my strengths and tell me I’m capable of taking leading positions. They were the ones that actually said the words… They’re really like big sisters. Anyone can tell you, “You’ll be successful, you’re great, you’re a leader,” but when someone that you have a good personal relationship with tells you that, you believe it, and that’s exactly what they do.* (Student 24)

The Ulpana emphasizes teamwork in all aspects, especially regarding social activities. This is highly noticeable in the holidays, ceremonies, and festivals organized by the students. It is the girls’ responsibility to write and direct school plays, decorate the sets, make costumes, arrange donations for charities, and produce evening activities. Not only do the Ulpana encourage every single student to actively participate and contribute her talent and skills to many of the school’s operations, but some of the key roles in these big productions are given to girls as a result of an election process. Student 21, for instance, referred to her election as the school play’s producer:

*I’m not sure someone directly told me I could lead. No one from the staff picked me for this role. My class did. The fact that the whole class picked me for this tells me a lot about how I should think of myself.*

Therefore, social persuasion regarding Ulpana girls comes from three sources: teachers and other adult members of staff, young women who volunteer as guides, and peers and classmates. In one way or another, all of these figures expressed their belief in the interviewees’ ability to lead to them.

### 5.4 Physical and emotional state

In order to find out whether the subjects of this research felt happy, confident, and free to speak their minds and had a generally good feeling during their day at school, they were asked questions about their emotional state. They were allowed to consider their social status in class, their contentment with the school’s endeavors for their success, the general school atmosphere, and their sense of well-being. The interviews revealed that both groups faced similar circumstances regarding their feelings while attending school.

#### 5.4.1 Girls attending secular schools

Most of the girls studying in the public education system (64.2%) expressed a positive attitude to school and their social status there.

*I like being at school with my friends. We have a special place where we all sit and share whatever we like. We also play there. I have about 15 or 16 friends.* (Student 5)

*I love coming to school, not so much for studying but mainly for the good mood I get there. When I was on the student council, I felt connected, and it was really fun being there.* (Student 12)

Four interviewees (28.5%) expressed that they felt negative emotions and were generally sad at school. For example, they talked about school being a boring place that plays no significant role in their lives or about it being unbearable socially:

*School isn’t that central or meaningful to me. Going there and sitting on my ass for 6 to 8 hours isn’t that important to me. Not as much as other things.* (Student 2)

*This whole concept of “friendship” is hypocritical. This whole game. So I’d rather not get into it, even if it means staying alone.* (Student 9)

Analyzing the interviews of girls studying in religious education institutions revealed a comparable situation.

#### 5.4.2 Girls attending Ulpana

Most of the girls (66.6%) indicated that they have a good time during school hours and look forward to meeting their friends every day. Most of them usually sleep there due to the boarding school conditions, which adds to their perceived sense of closeness and intimacy and their generally positive emotions regarding school.

*What I like most about this school is the attitude. It’s not just about studying. We have a lot of lectures, activities, social experiences. Not just learning. So it’s fun!* (Student 22)

The first reason is my friends. They share my lifestyle. They’re religious, and we basically think alike. The atmosphere is fun and good. We share stuff, you can say whatever you like, and you’ll be accepted. Everyone knows everyone on a deep level. Deep friendship. We really feel comfortable. I look forward to seeing them at school every day. (Student 26)

The other Ulpana students talked about school as a place they do not enjoy being. By using phrases like “I do not feel I belong” and “I cannot find my place,” they expressed how uncomfortable they sometimes feel.

*I don’t feel connected to school. I don’t really belong. I don’t know exactly how to explain it. I enjoy being there, but it’s not my place. (Student 20)*

*At the end of 11th grade, I felt really down. I still had my friends, but I couldn’t find my true self. It affected both my grades and my social status. (Student 24)*

### 5.5 Summary

Table 1 combines all four elements that affect the development of leadership self-efficacy and the differences between the two groups based on the extent to which the interviewees perceived them as meaningful. The numbers indicate the percentage of girls whose experiences reflected these factors.

**Table 1:** Factors contributing to leadership self-efficacy.

Factors contributing to leadership self-efficacy	Secular education *N* = 14		Religious education *N* = 12	
Mastery experiences	At school 78.5%	After school 14.2%	At school 66.6%	After school 100%
Social modeling at school	42.8%		91.6%	
Social persuasion of leadership ability	35.7%		83.3%	
Good physical and emotional state	64.2%		66.6%	

## 6 Discussion

This study has examined the effect post-primary school education has on female teenagers’ leadership self-efficacy using the theoretical framework of SCT, focusing on their personal experiences in both secular and religious middle and high schools. The data for the analysis was gathered using 26 in-depth interviews, which enabled a good understanding of the extent to which school influences these girls (according to their own perceptions) to believe in their ability to lead. The findings show that the establishment of leadership self-efficacy differs between the two educational sectors.

Since mastery experiences are considered to be effective in developing self-efficacy (Bandura, 1999, 2006), being able to master and perform as a leader in real-life experiences has a substantial value in developing leadership among teenage girls. Occupying a formal leadership position might serve as a key element in self-efficacy (Bussey and Bandura, 1999). In this regard, most of the girls from both groups in this study presented themselves as having high levels of guiding, controlling, and decision-making experience. It appears that adolescent girls at religious schools acquired much more experience while practicing leadership through formal education at school and non-formal youth organizations after school hours. However, it is not only the number of activities that affects these girls’ leadership experience but also their essence. Examining only the leadership experiences that take place at school and the roles girls play in them uncovers a much more independent and self-reliant approach in the religious schools. Girls are given almost complete autonomy by the Ulpana staff to lead projects. They are taught to trust themselves and strive for the goals they set for themselves. On the other hand, leadership experiences in the public secular education system involve adult assistance, instructions, and guidance during all stages of their assignments. Though this approach might lead to positive leadership outcomes (Van Linden and Fertman, 1998), it can, by contrast, result in low self-efficacy, an over-reliance on adults, and weakened self-confidence.

Social models such as peers or other significant individuals who succeed as leaders and are viewed by girls as similar to them can increase their self-efficacy (Bussey and Bandura, 1999) and, as a result, enhance their leadership development (Eva et al., 2021). Findings from this research indicate a significant difference between the two groups. Adults act as important role models for adolescent girls during the leadership development process (Zeldin et al., 2016; Fulton et al., 2019; Archard, 2012b), yet female students from secular institutions find it difficult to see teachers beyond their official profession. However, students from religious schools perceive their teachers as mentors and role models and speak about them with admiration. In correlation with studies that highlight the positive influence mentoring and role modeling has on student leadership behavior, self-regulation, relational development, self-understanding, and preparation for future leading roles (Archard, 2012a; Deutsch et al., 2017), this research suggests that religious public schools in Israel offer a more supportive environment for facilitating leadership development opportunities through mentoring compared to secular public schools.

Van Linden and Fertman (1998) emphasized that the first step in leadership development among teenagers is to have a significant adult make them aware of their leadership potential. When peers or adults who are considered role models express their faith in a teenager’s capability to achieve success and become a leader, self-efficacy is intensified (Bussey and Bandura, 1999). Still, the information gathered by this study’s interviews points out the negative effect secular education has on these girls’ self-efficacy: most of them had almost never experienced social persuasion regarding their leading abilities from their teachers or any other member of staff. In contrast, the religious group described a very positive approach. There are three sources of social persuasion in the religious schools’ environment: teachers and other adult members of staff, young female guide volunteers who are stationed at a school for one or two years, and peers and classmates. Consequently, leadership self-efficacy is more likely to be enhanced in these schools.

The fact that leadership self-efficacy seems more enhanced in the all-girl post-primary religious schools is somewhat surprising. One might assume that secular mixed-gender schools are more liberal, more equality-oriented, and probably more inclined to promote girls’ empowerment in a way that influences their day-to-day experiences. Religious schools, on the other hand, are usually perceived to emphasize traditional gender expectations and transmit hidden and implicit messages that prohibit the undermining of social constructions. Reflecting on possible explanations for this paradox suggests that a single-sex environment has the potential to increase leadership self-efficacy. This assumption correlates with the study of Shapiro et al. (2015), who argue that single-sex environments increase girls’ self-confidence, broaden their career options, and diminish gendered messages. Such environments enable girls to feel free from gender-based leader stereotypes since many girls believe they would act differently if boys were present (Whittington and Aspelmeier, 2018). An all-girls school also induces girls to fill all leadership roles (Archard, 2011, 2012b), an element the girls at religious schools talked about repeatedly when referring to the many projects they assigned themselves to lead. This liberating environment, where all leading positions, teachers, educators, advisors, and students are women, enables teenage girls to explore the extent to which they can develop their leadership potential.

## 7 Study limitations

The findings were drawn from a rather small sample of interviewees. A larger sample size might have revealed more details about how leadership skills in the two research groups are developed. Moreover, an additional perspective from the point of view of the school leadership team and educators could have provided insights into their level of commitment to and understanding of the problem. The data collection was also done at a single point in time and focused on the Israeli case study in particular. Adolescent girls were the primary concern, and the study was created for a specific age range. In this regard, the results may be somewhat limited in terms of time and location, even though they reveal significant new findings.

Additionally, although the schools involved in this study belong to one national educational system, one cannot ignore the worldwide cultural differences between secular and religious societies (Scheer, 2022; Beyers, 2017). This is, perhaps, more so particularly in the Israeli case study (Cooperman, 2016). The effect of religion on cultural socialization in childhood has been investigated elsewhere, with previous research examining various dimensions of religiousness or secularity in the adolescent’s family or social environment (Voas and Storm, 2019; Massengill and MacGregor, 2012; Sherkat, 2003). However, we referred in this study to the Israeli educational system as belonging to one socio-political entity, leaving room for further investigations into the specific cultural differences not only between secular and religious societies but also between different categories of religion (Cooperman, 2016).

## 8 Conclusion

Girls often have lower self-efficacy in their leadership abilities and tend to reinforce this type of behavior among one another (Weissbourd et al., 2015). Some of them are inclined to develop an incorrect concept of leadership through classroom-based experiences in a way that can sometimes damage their leadership potential (Wang and Wang, 2021). As educators and researchers who support leadership learning and development for young women, we should be aware of the social mechanisms that are usually unintentional and hard to detect and make it our goal to promote high efficacy known to provide positive guides for performance (Bandura, 1999). Exploring the extent to which girls believe in their ability to lead and their acquisition of the necessary means to achieve their leadership goals is of the highest importance. School has a considerable effect on character building and on creating a solid foundation for leadership among the youth, particularly young girls. Whilst this study is limited by its research sample size, it emphasizes the need to ensure that policymakers within the educational establishment in Israel acknowledge the importance of intensifying leadership self-efficacy by providing many more leadership experiences at school, social modeling and mentorship from leading figures, increased social persuasion of leadership capabilities from peers and teachers, and a peaceful, pleasant, and healthy school environment.

## Data Availability

The original contributions presented in the study are included in the article/supplementary material, further inquiries can be directed to the corresponding author.
